# Molybdenum cofactor deficiency: A natural history

**DOI:** 10.1002/jimd.12488

**Published:** 2022-03-03

**Authors:** Ronen Spiegel, Bernd C. Schwahn, Liza Squires, Nils Confer

**Affiliations:** ^1^ Emek Medical Center Afula Israel; ^2^ Rappaport school of Medicine Technion Haifa Israel; ^3^ Manchester Centre for Genomic Medicine, St Mary's Hospital Manchester University NHS Foundation Trust, Health Innovation Manchester Manchester UK; ^4^ Division of Evolution & Genomic Sciences, School of Biological Sciences, Faculty of Biology, Medicine and Health University of Manchester Manchester UK; ^5^ Origin Biosciences San Francisco California UK

**Keywords:** molybdenum cofactor deficiency, natural history study, neurodegenerative disorder, sulfite intoxication syndrome, sulfite oxidase deficiency

## Abstract

Molybdenum cofactor deficiency (MoCD) includes three ultrarare autosomal recessive inborn errors of metabolism (MoCD type A [MoCD‐A], MoCD‐B, and MoCD‐C) that cause sulfite intoxication disorders. This natural history study analyzed retrospective data for 58 living or deceased patients (MoCD‐A, *n* = 41; MoCD‐B, *n* = 17). MoCD genotype, survival, neuroimaging, and medical history were assessed retrospectively. Prospective biomarker data were collected for 21 living MoCD patients. The primary endpoint was survival to 1 year of age in MoCD‐A patients. Of the 58 MoCD patients, 49 (MoCD‐A, *n* = 36; MoCD‐B, *n* = 13) had first presenting symptoms by Day 28 (neonatal onset; median: 2 and 4 days, respectively). One‐year survival rates were 77.4% (overall), 71.8% (neonatal onset MoCD‐A), and 76.9% (neonatal onset MoCD‐B); median ages at death were 2.4, 2.4, and 2.2 years, respectively. The most common presenting symptoms in the overall population were seizures (60.3%) and feeding difficulties (53.4%). Sequelae included profound developmental delay, truncal hypotonia, limb hypertonia that evolved to spastic quadriplegia or diplegia, dysmorphic features, and acquired microcephaly. In MoCD‐A and MoCD‐B, plasma and urinary xanthine and S‐sulfocysteine concentrations were high; urate remained below the normal reference range. MOCS1 mutation homozygosity was common. Six novel mutations were identified. MoCD is a severe neurodegenerative disorder that often manifests during the neonatal period with intractable seizures and feeding difficulties, with rapidly progressive significant neurologic disabilities and high 1‐year mortality rates. Delineation of MoCD natural history supports evaluations of emerging replacement therapy with cPMP for MoCD‐A, which may modify disease course for affected individuals.

## 1 INTRODUCTION

Excessive sulfite accumulation due to deficient sulfite oxidase (EC 1.8.3.1.) causes a distinctive infantile neurological syndrome that was first described in 1967.^1^ Sulfite (SO_3_^2−^) is a highly neurotoxic molecule^2^, ^3^, ^4^, ^5^ produced during catabolism of the sulfur amino acids methionine and cysteine and is normally oxidized rapidly by sulfite oxidase to nontoxic sulfate (SO_4_^2−^).^6^, ^7^ Sulfite reacts with cysteine to form S‐sulfocysteine (SSC), which has neuroexcitatory properties.^8^, ^9^, ^10^

Primary or isolated sulfite oxidase deficiency (ISOD) is caused by biallelic pathogenic variants in the *SUOX* gene. Sulfite oxidase deficiency can also arise secondary to genetic disorders that disrupt de novo synthesis of molybdenum cofactor (MoCo), which is required for sulfite oxidase function. MoCo is synthesized by a three‐step biosynthetic pathway that involves the products of four genes, *MOCS1*, *MOCS2*, *MOCS3*, and *GPHN*.^11^

Sulfite intoxication disorders are ultrarare, with published reports of about 100 patients with molybdenum cofactor deficiency (MoCD) and 50 with ISOD.^12^, ^13^ Biallelic mutations in MOCS1 result in MoCD type A (MoCD‐A), which is found in two‐thirds of patients and is estimated to occur in 1:341 690 to 1:411 187 births worldwide (Mayr et al., unpublished data, March 2020), though incidence is higher in some regions. Most other cases are MoCD‐B caused by MOCS2 or MOCS3 mutations. Only a few cases of MoCD‐C (caused by GPHN mutations) are currently known.^11^, ^12^, ^14^, ^15^, ^16^, ^17^

Clinical manifestation of the four distinct disorders is believed to be indistinguishable. Most published cases show classical presentation during the first postnatal days, including intractable seizures, feeding difficulties, severe encephalopathy, apneas, exaggerated startle reactions, and axial hypotonia and limb hypertonia.^2^, ^18^, ^19^ If the neonatal period is survived, infants continue to have myoclonic and generalized seizures and develop severe dystonic spastic cerebral palsy.^7^ Mortality is high due to intercurrent lower respiratory tract infections and seizures, with a reported median survival of 3 years.^16^ Generally, symptom onset is later in milder cases, and occasionally children present only with dystonia, spasticity, and variable degree of developmental delay.^20^, ^21^ Such cases are believed to represent attenuated disease owing to residual MoCo synthesis.

Although case reports and small cohort studies have described patients with MoCD and reported the associated genotypes, information remains limited regarding the disease natural history, associated biomarkers, and genotype–phenotype correlations.^12^, ^14^, ^16^ The metabolic block in MoCD‐A can be bypassed by replacing cyclic pyranopterin monophosphate,^18^, ^21^, ^22^ making it important to compare clinical outcomes of the treatment with disease natural history. A previous natural history study did not examine a genetically confirmed MoCD population or prospective data.^16^ We, therefore, conducted this study to characterize the natural history of patients with inherited sulfite intoxication disorders and provide a more complete understanding of the clinical, molecular and biochemical variability of the condition, with specific emphasis on MoCD in general, and MoCD‐A in particular.

## 2 METHODS

### 2.1 Study design and participants

This noninterventional, retrospective and prospective, observational, multinational, natural history study enrolled 65 patients with sulfite intoxication disorders, including MoCD‐A, MoCD‐B, and ISOD (ClinicalTrials.gov Identifier: NCT01735188). The country of residence for all patients enrolled in the study is listed in Figure S1. Living or deceased patients were enrolled if they had a genetic, clinical, and/or biochemical diagnosis of MoCD or ISOD. Biochemical diagnosis included high SSC levels in urine, serum, or plasma or ≥2 positive urine sulfite dipsticks.

Two of the 65 enrolled patients were excluded from analysis because insufficient data were available to determine MoCD type (Figure S2). Five patients, who were enrolled based on phenotype, were removed from the analysis after genetic confirmation of ISOD. Fifty‐eight patients had genetic confirmation of MoCD. Four patients died before study start without genetic testing but were considered to have probable MoCD‐A because younger siblings were enrolled with genetically confirmed MoCD‐A. One additional patient died before genetic testing, but was considered to have MoCD‐B because both parents were carriers of a familial disease‐causing variant in *MOCS2*. These five patients were included in analyses with the respective MoCD subtypes. In addition, the reported genotype for two patients was not consistent with the reported type of disease, and because the genotype could not be verified, the authors relied on the disease classification as reported by the investigators. The study population therefore included 41 patients with MoCD‐A and 17 with MoCD‐B.

Retrospective data were assessed for all 58 patients and included demographics, MoCD genotype, family and medical history, survival, disease onset and presenting symptoms, sequelae, and neuroimaging. Twenty‐one patients (MoCD‐A, *n* = 14; MoCD‐B, *n* = 7) were alive at enrollment and provided up to 12 months of prospective biomarker data, including plasma and urinary SSC, urate, and xanthine, collected at baseline and Months 3, 6, 9, and 12 (Supporting Information Methods S1).

The study was conducted according to Declaration of Helsinki and International Council for Harmonization guidelines. Institutional review boards at each study site approved the study protocol and informed consent forms. Parents or a legal guardian or representative provided written informed consent to participate.

### 2.2 Statistical analysis

The primary endpoint was survival to 1 year of age in patients with MoCD‐A. Survival was calculated using the Kaplan–Meier method and analyzed by year of birth and age of symptom onset. Secondary endpoints included changes in plasma and urinary biomarkers, survival rates, disease sequelae, and evaluations of brain magnetic resonance imaging (MRI) abnormalities. Data were assessed for the overall population according to disease subtype (MoCD‐A and MoCD‐B) and neonatal onset (≤28 days), and for living patients. Continuous data were analyzed using descriptive statistics. Categorical data were summarized using frequencies and descriptive statistics.

## 3 RESULTS

### 3.1 Participant characteristics

In the overall population (*N* = 58), 72.4% were male and the first signs of MoCD were documented at a median 2 days of age (range, 1–927) (Table 1). MoCD symptoms were documented in the neonatal period for 36 patients with MoCD‐A and 13 with MoCD‐B (median, 2 and 4 days, respectively). Seven patients with disease symptoms beginning after the neonatal period had first symptoms recorded at a median 218 days (range, 46–927).

**TABLE 1:** Patient characteristics

Characteristic	MoCD type A	MoCD type B	Total
Sex, *n* (%)			
*n*	41	17	58
Male	32 (78.0)	10 (58.8)	42 (72.4)
Female	9 (22.0)	7 (41.2)	16 (27.6)
Parental consanguinity, *n* (%)			
*n*	41	17	58
Yes	28 (68.3)	9 (52.9)	37 (63.8)
No	12 (29.3)	7 (41.2)	19 (32.8)
Unknown	1 (2.4)	1 (5.9)	2 (3.4)
Gestational age at birth, weeks			
n	32	11	43
Mean (*SD*)	39.1 (1.2)	38.3 (2.6)	38.9 (1.7)
Birth weight, kg			
*n*	22	10	32
Mean (*SD*)	3.5 (0.3)	3.3 (0.4)	3.4 (0.3)
Head circumference at birth, cm			
*n*	14	6	20
Mean (*SD*)	34.4 (0.9)	35.0 (1.9)	34.6 (1.3)
MoCD symptoms documented at any time, *n* (%)			
*n*	41	17	58
Seizures	38 (92.7)	16 (94.1)	54 (93.1)
Feeding difficulty	35 (85.4)	13 (76.5)	48 (82.8)
High‐pitched cry	16 (39.0)	6 (35.3)	22 (37.9)
Exaggerated startle response	14 (34.1)	5 (29.4)	19 (32.8)
Metabolic acidosis	7 (17.1)	3 (17.6)	10 (17.2)
Intracranial hemorrhage	3 (7.3)	2 (11.8)	5 (8.6)
Other^a^	13 (31.7)	8 (47.1)	21 (36.2)
No symptoms recorded	0	1 (5.9)	1 (1.7)
Age at first documented symptom, days			
*n*	41	16	57
Median (range)	2 (1–927)	6 (1–218)	2 (1–927)
Status at study end, *n* (%)			
*n*	41	17	58
Alive	16 (39.0)	7 (41.2)	23 (39.7)
Deceased	25 (61.0)	10 (58.8)	35 (60.3)
Age at death, days			
*n*	25	9	34
Median (range)	864 (10–4537)	918 (11–4360)	870 (10–4537)

Abbreviations: MoCD, molybdenum cofactor deficiency; *SD*, standard deviation.

^a^ Other included total number of patients with age of onset listed for a symptom, not the total number of other symptoms.

The most common presenting symptoms in the overall population were seizures (60.3%) and feeding difficulties (53.4%), which first occurred at a median of 2 (range, 1–733) and 2 (range, 1–218) days of age, respectively. The most common symptoms reported at any time were seizures (overall, 93.1%; MoCD‐A, 92.7%; MoCD‐B, 94.1%) and feeding difficulties (overall, 82.8%; MoCD‐A, 85.4%; MoCD‐B, 76.5%). Although no formal comparisons were made, similar trends in presenting signs and symptoms were observed between MoCD subtypes.

### 3.2 MoCD sequelae

MoCD sequelae were reported for 93.1% (54/58) of patients. The most common sequelae were limb hypertonia (84.5%), spastic quadriplegia (56.9%) or diplegia (24.1%), severe global developmental delay (81.0%), truncal hypotonia (74.1%), dysmorphic features (67.2%), and acquired microcephaly (63.8%). Ophthalmological sequelae were common in both subtypes and included cerebral blindness, ectopic lenses, and nystagmus (Table S1). Sequelae from patients with neonatal onset MoCD were similar in nature but occurred at an earlier stage of life and generally culminated in more severe disability when compared to patients with postneonatal onset. Data on sequelae were unavailable for two patients with MoCD‐A and two with MoCD‐B.

### 3.3 Biochemical markers

In patients followed prospectively, levels of SSC, xanthine, and urate remained unchanged over 12 months. Median urine SSC and xanthine concentrations were persistently increased (≥5.7‐fold and ≥4.5‐fold, respectively) above the upper limit of normal (reference ranges for 0–1 year of age: 0–18 mmol/mol creatinine [SSC], 0–63.4 mmol/mol creatinine [xanthine])^22^ throughout the prospective data collection period (Figure 2A,B). Urine urate concentrations remained below the 95% confidence interval (CI) for the reference range (820–1026 mmol/mol creatinine)^22^ (Figure 2C). Median plasma concentrations of SSC and xanthine remained similarly increased over 1 year at ≥2.3‐fold and ≥1.5‐fold above the upper limit of normal of 3 and 7 μmol/L, respectively^22^ (Figure 2D,E), and plasma urate concentrations remained below the 95% CI for the lower limit of normal (120 μmol/L for infants and children, Figure 2F).^22^, ^23^

### 3.4 Neuroimaging findings

Thirty‐five patients with MoCD‐A had MRI results, which included abnormal white matter (71.4%), cortical atrophy (68.6%), abnormal corpus callosum (54.3%), cyst formation (45.7%), abnormal basal ganglia (40.0%), and hydrocephalus (34.3%). Fifteen of sixteen patients with MoCD‐B and available brain MRI results had abnormal findings, including cortical atrophy (68.8%), abnormal white matter (62.5%), abnormal basal ganglia (50.0%), and abnormal corpus callosum (43.8%). Of these 16 patients, 1 patient with MoCD‐B had no abnormalities reported in an MRI obtained when the patient was approximately 5 years old. One additional patient with MoCD‐B did not have an MRI performed. Findings for patients with neonatal onset of MoCD‐A and MoCD‐B were consistent with those of the overall MoCD‐A and MoCD‐B groups.

### 3.5 Survival

**FIGURE 1:**
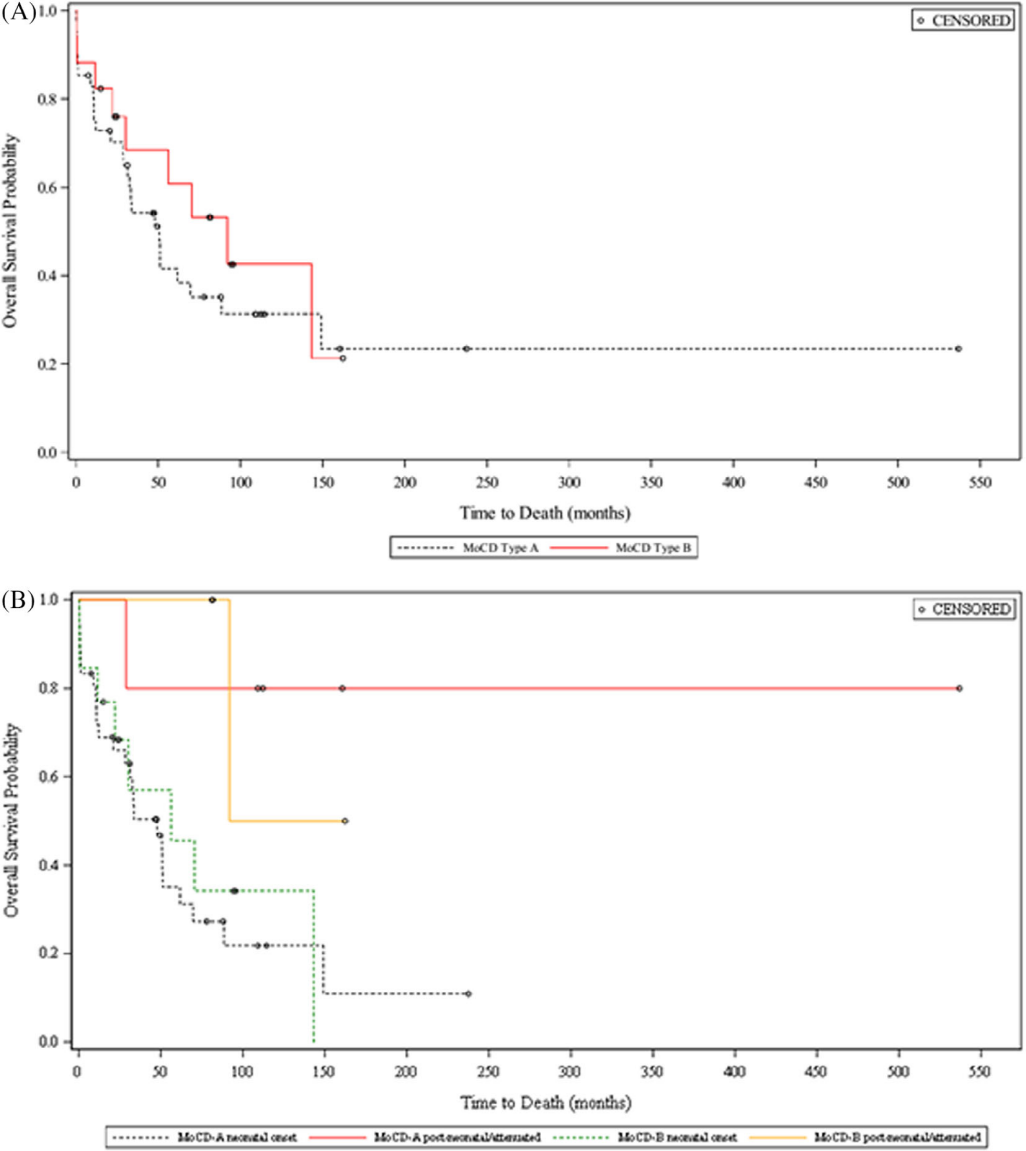
Kaplan–Meier estimates of survival probability. (A) Full analysis by MoCD type (in the full analysis by MoCD type, MoCD‐A *n* = 41 and MoCD‐B *n* = 17). (B) Neonatal onset and postneonatal onset by MoCD type (neonatal onset was defined as patients with onset of MoCD by 28 days. Postneonatal onset was defined as patients with onset of MoCD symptoms beyond 28 days of postnatal age. MoCD‐A neonatal onset *n* = 36, MoCD‐A postneonatal onset/attenuated *n* = 5, MoCD‐B neonatal onset *n* = 13, and MoCD‐B postneonatal onset/attenuated *n* = 4)

The 1‐year survival was 77.4% (MoCD‐A, 75.3%; MoCD‐B, 82.4%) (Figure 1). In MoCD‐A, survival was further reduced to 35.1% (neonatal onset) and 41.6% (all MoCD‐A patients) at 5 years of age. The median survival time was 4.23 (MoCD‐A; 3.98 years with neonatal onset) and 7.66 years (MoCD‐B). The median age at death in the overall population was 2.4 years (range, 10 days to 12.4 years). Patients with MoCD‐A and MoCD‐B had median ages at death of 2.4 and 2.5 years, respectively. Among patients with neonatal onset, 71.8% with MoCD‐A survived to 1 year of age (median age at death, 2.4 years) and 76.9% with MoCD‐B survived to 1 year (median age at death, 2.2 years). During the prospective data collection period, three patients died: one patient with MoCD‐A (5.1 years old) reportedly due to sepsis and intracranial hemorrhage, and two patients with MoCD‐B reportedly due to pneumonia (2.5 years old) and lung infection (5.9 years old).

Analysis of survival by birth year showed that the following patients survived to 1 year of age: 18/21 (85.7%) patients born in or after 2010, 25/34 (73.5%) born during 2000–2009, and 5/7 (71.4%) born during 1990–1999. Among deceased patients, median ages at death were 1.8, 2.6, and 2.6 years, respectively.

### 3.6 Genotypes

Consistent with the high consanguinity rate, most patients had homozygous pathogenic mutations (Table 2 and Figure S3). Generally, mutations were private or shared by individuals from the same ethnic group apart from three variants that were found in a few individuals from different ethnic groups, in particular c.217C>T in *MOCS1* which was found in three ethnic groups. The low allele frequency of variants in *MOCS1* does not suggest the presence of multiple mutational hot spots. Most patients' diagnosis was ascertained during the neonatal period, suggesting their genotype confers severe disease. Nevertheless, a few variants in *MOCS1* and *MOCS2* were associated with postneonatal symptom onset and/or longer survival. Six novel genetic variants were identified, including three in *MOCS1* and three in *MOCS2*.

**TABLE 2:** *MOCS1* and *MOCS2* pathogenic variants identified in study cohort

Gene/disrupted isoform	Allele	Protein	Number of alleles	Mutation type	Number of patients with mutation	Patient identification number	Ethnicity/race^a^	Previous reports of mutation
MoCD‐A								
*MOCS1*/MOCS1A	c.99_100delGG^b^	p.Glu34ValfsTer154	2	Frame shift	1	502A11	Asian (*n* = 1)	Reiss et al.^11^
*MOCS1*/MOCS1A	c.217C>T	p.Arg73Trp	10	Missense	5	502A28 502A29 502A37 502A38 502A40^c^	White (*n* = 2) White: Hispanic/Latino (*n* = 1) Other (*n* = 2)	Reiss et al.,^24^ Gumus et al.^25^
*MOCS1*/MOCS1A	c.251_418del	p.Cys84fs	2	Frame shift	1	502A15	White (*n* = 1)	Carmi‐Nawi et al.^26^
*MOCS1*/MOCS1A	c.256T>G	p.Tyr86Asp	1	Missense	1	502A8	White (*n* = 1)	Reiss et al.^11^
MOCS1/MOCS1A	c.367C>T	p.Arg123Trp	6	Missense	3	502A35 502A19^d^ 502A20^d^	White (*n* = 3)	Reiss et al.^27^
*MOCS1*/MOCS1A	c.377G>A	p.Gly126Asp	1	Missense	1	502A41^e^	White (*n* = 1)	Reiss et al.,^24^ Mills et al.^28^
*MOCS1*/MOCS1A	c.394C>T	p.Arg132Trp	2	Missense	2	502A24 502A25	Asian (*n* = 2)	Reiss et al.^11^
*MOCS1*/MOCS1A	c.418+1G>A	Splice site	2	Splice site	1	502A26	White (*n* = 1)	Reiss et al.,^24^ Mills et al.^28^
*MOCS1*/MOCS1A	c.586A>G	p.Phe196Val	2	Missense	1	502A21	White (*n* = 1)	Novel
*MOCS1*/MOCS1A	c.583+1G>A^f^	Splice site	1	Splice site	1	502A5	Asian (*n* = 1)	Leimkuhler et al.^29^
*MOCS1*/MOCS1A	c.645+2T>G	Splice site	4	Splice site	2	502A32 502A33	White (*n* = 2)	Novel
*MOCS1*/MOCS1A	c.757+1G>C	Splice site	3	Splice site	2	502A30 502A31	Other (*n* = 2)	Novel
*MOCS1*/MOCS1A	c.949C>T	p.Arg317Cys	2	Missense	1	502A27	Asian (*n* = 1)	Yoshimura et al.^30^
*MOCS1*/MOCS1A	c.956G>A	p.Arg319Gln	2	Missense	1	502A10	Asian (*n* = 1)	Reiss et al.^24^
*MOCS1*/MOCS1A	c.970G>A	p.Gly324Arg	1	Missense	1	502A9	White: Hispanic/Latino (*n* = 1)	Reiss et al.^27^
*MOCS1*/MOCS1A	c.971G>A	p.Gly324Glu	6	Missense	3	502A16 502A17 502A18	White (*n* = 3)	Reiss et al.,^24^ Zaki et al.^12^
*MOCS1*/MOCS1A	c.1000dupT^g^	p.Gly334PhefsTer2	2	Frame shift	2	502A24 502A25	Asian (*n* = 2)	Reiss et al.^11^
*MOCS1*/MOCS1A	c.1015_1018delCGGG^h^	p.Arg339IlefsTer13	1	Frame shift	1	502A14	Other (*n* = 1)	Leimkuhler et al.^29^
*MOCS1*/MOCS1A	c.1102+1G>A	Splice site	3	Splice site	2	502A34 502A41^e^	White (*n* = 2)	Reiss et al.,^24^ Zaki et al.^12^
*MOCS1*/MOCS1A	c.1150G>A	p.Glu384Lys	3	Missense	3	502A8 502A9 502A14	White (*n* = 1) White: Hispanic/Latino (*n* = 1) Other (*n* = 1)	Reiss et al.^24^
*MOCS1*/MOCS1AB	c.1165+6T>C^i^	Splice site	2	Splice site	1	502A12^e^	Asian (*n* = 1)	Arenas et al.^31^
*MOCS1*/MOCS1AB	c.1338delG	p.Arg447AspfsTer31	2	Frame shift	1	502A6	Asian (*n* = 1)	Mayr et al.^20^
*MOCS1*/MOCS1AB	c.1508_1509delAG	p.Glu503AspfsTer16	4	Frame shift	2	502A39 502A7^j^	White (*n* = 2)	Reiss et al.^24^
*MOCS1*/MOCS1AB	c.1643C>A	p.Ala543Glu	4	Missense	2	502A22 502A23	Asian (*n* = 2)	Higuchi et al.^32^
*MOCS1*/MOCS1AB	c.1762G>A^k^	p.Gly588Arg	2	Missense	1	502A13	Other (*n* = 1)	Leimkuhler et al.,^29^ Veldman et al.^33^
MoCD‐B								
*MOCS2*/*MOCS2A*	c.3G>A	p.Met1Ile	6	Missense	3	502B10 502B14^e^ ^,^ ^l^ 502B17	White (*n* = 3)	Reiss et al.,^34^ Zaki et al.^12^
*MOCS2*/MOCS2A	c.57A>T	p.Leu19Phe	2	Missense	1	502B6^e^	White: Hispanic/Latino (*n* = 1)	Novel
*MOCS2*/MOCS2A	c.‐48+1G>A^m^	Splice site	2	Splice site	1	502B11	White (*n* = 1)	Novel
*MOCS2*/*MOCS2A*	c.226G>A	p.Gly76Arg	8	Missense	4	502B4^n^ 502B5^n^ 502B8^n^ 502B9	Asian (*n* = 4)	Mills et al.,^28^ Reiss et al.^27^
*MOCS2*/*MOCS2A*	c.252_253InsC^o^	p.Lys85GlnfsTer5	2	Frame shift	1	502B13	Other (*n* = 1)	Reiss et al.^34^
*MOCS2*/*MOCS2A*	c.314delA	p.Lys105ArgfsTer15	2	Frame shift	1	502B15	White (*n* = 1)	Novel
*MOCS2*/*MOCS2B*	c.377+1G>A^p^	Splice site	1	Splice site	1	502B2	Other (*n* = 1)	Reiss et al.^11^
*MOCS2*/*MOCS2B*	c.471_477delTTTAAAAinsG^q^	p.Leu158_Lys159del	2	Frame shift	1	502B16	White (*n* = 1)	Reiss et al.^27^
*MOCS2*/MOCS2B	c.539_540delAA	p.Lys180ArgfsTer31	3	Frame shift	2	502B2 502B12	White (*n* = 1) Other (*n* = 1)	Reiss et al.,^34^ Hinderhofer et al.^35^
*MOCS2*/MOCS2A	c.‐9_14del23^r^	p.(0)	2	Initiation failure	1	502B7	Black/African American (*n* = 1)	Hahnewald et al.^36^

^a^ Except where otherwise specified, patient ethnicity was reported as “not Hispanic/Latino.”

^b^ This allele was recorded as c.99_100Del but recorded here as c.99_100delGG.

^c^ For one patient, the allele was recorded as c.271C>T but recorded here as c.217C>T.

^d^ For two patients, this allele was recorded as c.1660C>T but recorded here as c.367C>T.

^e^ Patients with attenuated progression.

^f^ This allele was recorded as c.589+1 G>A and is listed in this table as c.583+1G>A.

^g^ This allele was recorded as c.1000insT but recorded here as c.1000dupT.

^h^ This allele was recorded as c.1015_1018del4 but recorded here as c.1015_1018delCGGG.

^i^ This allele was recorded as G.IVS9+6T>C and based on new nomenclature, is listed in this table as c.1165+6T>C.

^j^ This allele was recorded as c.1523‐delAG and based on new nomenclature, is listed in this table as c.1508_1509delAG.

^k^ This allele was recorded as c.1777G>A and based on new nomenclature, is listed in this table as c.1762G>A.

^l^ This patient carries an additional variant of c.1667G>A in *MOCS1*.

^m^ This allele was recorded as c.140+1G>A and based on new nomenclature, is listed in this table as c.‐48+1G>A.

^n^ This allele was recorded as c.413G>A and based on new nomenclature, is listed in this table as c.226G>A.

^o^ This allele was recorded as C.252dupC and based on new nomenclature, is listed in this table as c.252_253InsC.

^p^ This allele was recorded as c.564+1G>A and based on new nomenclature, is listed in this table as c.377+1G>A.

^q^ This allele was recorded as c.658_664delTTTAA AAinsG and based on new nomenclature, is listed in this table as c.471_477delTTTAAAAinsG.

^r^ This allele was recorded as a deletion of 23 BP (DEL‐9(23)) in the first exon of MOCS2A gene but recorded here as c.‐9_14del23.

### 3.7 Postneonatal onset

**FIGURE 2:**
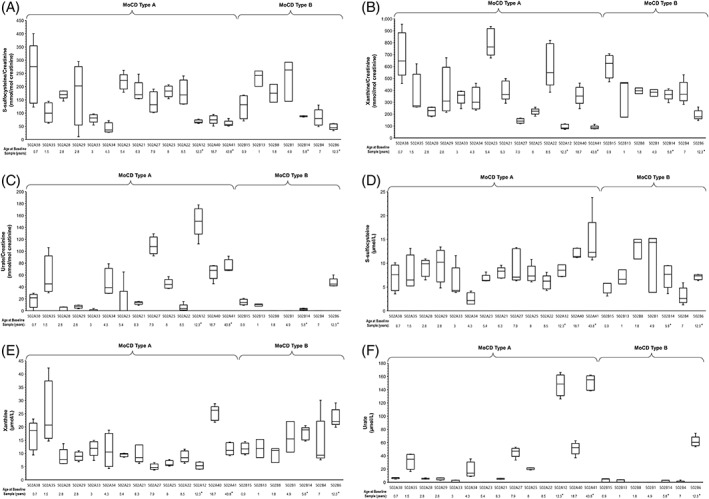
Prospectively collected biomarker concentrations in (A–C) urine and (D–F) plasma over the course of 1 year, representing 3–5 samples each for (A and D) S‐sulfocysteine, (B and E) xanthine, and (C and F) urate. (A) S‐sulfocysteine levels in urine. (B) Xanthine levels in urine. (C) Urate levels in urine. (D) S‐sulfocysteine levels in plasma. (E) Xanthine levels in plasma. (F) Urate levels in plasma. MoCD‐A, molybdenum cofactor deficiency type A. Box plots show maximum, third quartile, median, first quartile, and minimum. *Asterisks indicate patients with attenuated progression. Plasma reference ranges (all ages) are: 0–3 μmol/L (S‐sulfocysteine), 0–7 μmol/L (xanthine), and lower limit of normal of 120 μmol/L for infants and children (95% CI) (urate).^22^, ^23^ Urine reference ranges (0–1 year of age) are: 0–18 mmol/mol creatinine (S‐sulfocysteine), 0–63.4 mmol/mol creatinine (xanthine), 820–1026 mmol/mol creatinine (95% CI) (urate)^22^

Seven patients (four MoCD‐A [502A12, 502A24, 502A27, 502A41], three MoCD‐B [502B10, 502B14, 502B17]) had postneonatal onset of symptoms. Although these patients had a variable disease presentation and clinical course, the patients were generally characterized by later symptom onset (46–927 days after birth) and longer survival in most (Figure 1B). In addition to these seven patients, one patient with MoCD‐A (502A6) was documented in the study database as having symptoms at Day 1 of age but had longer survival, more in agreement with postneonatal onset patients, and one patient with MoCD‐B (502B6) had sequelae reported as absent at all retrospective and prospective data collection points, had milder biochemical abnormalities and longer survival. Of these patients, four patients had prospective biomarker collection characterized by relatively milder biochemical abnormalities, especially plasma and urinary urate (Figure 2). Several mutations were associated with postneonatal onset and/or longer survival, including c.1338delG, c.1165+6T>C, c.377G>A, c.949C>T, c.394C>T, c.1000dupT, and c.1102+1G>A in *MOCS1* and c.3G>A, and c.57A>T in *MOCS2* (Table 2).

## 4 DISCUSSION

### 4.1 Participant characteristics

This multinational retrospective and prospective study of inherited sulfite intoxication disorders reports the largest cohort to date of patients with genetically confirmed MoCD. A previous literature review summarized 82 MoCD patients with emphasis on clinical and radiological features.^16^ However, only 30% were genetically confirmed, precluding categorization according to MoCD subtype. In the current study 92% of cases had genetic confirmation of MoCD (*n* = 53). Five individuals without genetic results had a sibling with genetic confirmation or both parents with a pathogenic variant. Whereas previous studies report the disease affects both sexes equally,^16^ in the current study 78% of patients with MoCD‐A were male, while 59% with MoCD‐B were male. The high proportion of male MoCD‐A patients is not currently explained and warrants attention in future studies.

### 4.2 Clinical and biochemical presentation

MoCD‐A and MoCD‐B overlapped in their clinical and biochemical features and were primarily distinguished only by genetic assessment. Consistent with a diagnosis of MoCD, most patients presented with first disease symptoms within 28 days of birth, at a median age of 2 days. This is in accordance with the previous study by Mechler et al.^16^ Although most patients presented with multiple symptoms, seizures and feeding difficulties were most common. Neonatal presentation was generally associated with a more homogeneous and severe phenotype than postneonatal onset.

Seizures were the most common MoCD symptom, followed by feeding difficulties, both of which are early signs of acute encephalopathy. Seizures and feeding problems were also the most common presenting symptoms in the Mechler study, but were reported at lower frequencies probably due to the literature review study design.^16^ MoCD sequelae occurred for nearly all patients, with limb hypertonia most frequent, and gradually evolved to spastic quadriplegia or diplegia, severe global developmental delay, truncal hypotonia, acquired microcephaly, and dysmorphic features.

Prospectively collected biochemical profiles were similar between MoCD‐A and MoCD‐B and did not permit differentiation. Biochemistry showed persistently elevated urinary SSC and xanthine concentrations with some interpatient variability. With the exception of patients with postneonatal onset and longer survival, extremely low urate concentrations were observed. Levels of SSC, xanthine, and urate remained stable in individual patients over 12 months. Importantly, plasma urate concentrations can be normal in the first postnatal days before they decrease. Given this, low or decreasing urate can help increase suspicion of MoCD,^37^ while SSC and xanthine are best suited for biochemical diagnosis as levels are elevated at birth or increase soon after.^38^

Due to the progressive nature of symptoms in neonatal onset MoCD, rapid genetic confirmation is supported in suspected cases, as illustrated by the recent report by Kingsmore et al.^39^ These findings suggest pediatricians should have a high index of suspicion and obtain supportive biochemical data, such as urinary and blood SSC, xanthine, and urate, and urinary sulfites, in neonates who present with seizures and even subtle signs of encephalopathy.

Serum and urinary SSC, xanthine, and urate may also serve as reliable biomarkers for disease‐modifying treatments in both MoCD subtypes. For example, in preclinical studies in murine models and patients with MoCD‐A, the biosynthetic intermediate of MoCo, cPMP, has been shown to normalize disease biomarkers.^38^ This was further supported by normalization of these biomarkers in the few MoCD‐A patients who have received treatment with replacement cPMP.^33^, ^38^, ^40^ Now that replacement therapy with cPMP is approved in the United States for MoCD‐A^41^ and in development elsewhere, these data further emphasize the need for rapid diagnosis.

### 4.3 Neuroimaging findings

Neuroradiological abnormalities were often apparent within the first weeks of life. Consistent with previous studies,^14^, ^16^, ^42^ all but one patient with MoCD had abnormal MRI findings, with progressive cortical atrophy, abnormal white matter changes, and thinning of the corpus callosum being the most common. MRI scans are preferred over ultrasound, which can misinterpret early changes during the first days of life. Of interest, anecdotal reports describe abnormal fetal MRI findings in the third trimester, including global brain atrophy and cystic encephalomalacia,^26^, ^43^, ^44^ suggesting sulfite intoxication may initiate to some degree in utero.

### 4.4 Survival

Though statistical significance was not assessed for survival data, 1‐year survival rates were similar between MoCD‐B and MoCD‐A. In agreement with the authors' clinical experience, the median survival time was longer for patients with postneonatal onset compared to those who presented with disease‐specific symptoms in the neonatal period. The median survival time in this study was 4.23 years and was higher than the median survival of 36 months in Mechler's combined MoCD cohort.^16^ This may be related to improved standard of care.

### 4.5 Genotypes

Because retrospective data were included in this study, many molecular genetic investigations predated patient enrollment and reports often employed out‐of‐date nomenclature or reference sequences. The reported variants were thoroughly assessed for consistency and renamed according to current standards of the Human Genome Variation Society nomenclature for the description of sequence variants (www.hgvs.org/mutnomen) using cDNA RefSeq NM_001358530.2 (*MOCS1*) and NM_004531.5 (*MOCS2*). Two genetic reports (patient numbers 502A36 and 502B1) communicated by local investigators were inconsistent with the reported disease subtype. We were not able to verify the genetic data and therefore did not include them in Table 2, and classified the patients according to the reported disease type. We have included these patients in Table S2 where we provide more detail.

Genetic mutations were largely heterogeneous, mostly occurring in single families, and included nonsense, frameshift, splicing, and missense mutations (Table 2). Most affected the MOCS1A isoform; five disrupted the fusion MOCS1AB isoform. Also, most resulted in severe disease, though a few (*MOCS1*: c.1338delG, c.1165+6T>C, c.377G>A, c.949C>T, c.394C>T, c.1000dupT, and c.1102+1G>A; *MOCS2*: c.3G>A, c.57A>T) were associated with postneonatal onset or longer survival. This was exemplified by a patient (502A6) with a homozygous c.1338delG mutation whose relatively milder presentation was explained by an alternative initiation site, with partial transcription of MOCS1B allowing residual enzyme activity.^20^ Notably, c.1102+1G>A was previously reported as neonatal onset when in the homozygous state,^12^ and c.394C>T/c.1000dupT is associated with neonatal onset in another patient in this study. Three of the 25 *MOCS1* mutations were novel.

### 4.6 Postneonatal onset and attenuated progression

In addition to a need for rapid testing of patients with neonatal onset and classical presentation, it is also important to promptly identify those with postneonatal presentation so they (MoCD‐A patients) can receive cPMP replacement treatment as early as possible. Seven patients with symptom onset in the postneonatal period had a broader clinical phenotype than those with symptoms documented as neonates. While four patients progressed to severe disability akin to that seen with neonatal onset, three had attenuated disease progression, characterized by a broader spectrum of severity of disease sequelae, less pronounced abnormalities of purine biomarkers, and by longer survival. Importantly, we cannot exclude that patients with a postneonatal diagnosis may have developed MoCD symptoms before Day 28 that were not recorded in the medical history or not recognized as related to MoCD. For example, seizures may have occurred but were subclinical, transient, or unrecognized by parents. Although patients with later symptom onset may have attenuated disease, patients with attenuated disease may not always have a postneonatal presentation. One patient (502A6) with mild symptoms and biochemical profile was documented in the study database as having mild symptoms on Day 1. This variant (c.1338delG) was previously associated with attenuated disease.^20^ In this current analysis, patient 502A6 was considered to have neonatal onset and attenuated disease. In addition, one MoCD‐B patient (502B6) had sequelae reported as absent at all data collection points, had a milder biochemical abnormality and longer survival.

### 4.7 Study limitations

Study limitations were primarily consequences of the nature of MoCD and related to study design. Due to the low incidence of MoCD, the sample size was necessarily small, but to our knowledge this is the largest population of genetically confirmed MoCD patients published to date. The study used a combined retrospective and prospective approach to increase the sample size, and though the retrospective portion permitted inclusion of additional patients, some information was missing due to lack of systematic documentation. For example, confirmatory genetic analysis was unavailable for 5 deceased patients though genotype was inferred with a high degree of confidence based on evaluations of affected siblings or living parents. Prospectively collected clinical and biochemical data were available for the prospective cohort, but the data on clinical characteristics were not particularly informative. While biomarker data showed limited intra‐ and some interindividual variability, especially in urine biomarkers, there was no discernible trend over the prospective observation period of 12 months. Finally, little is known about the effect of diet on MoCD‐related biomarkers,^45^, ^46^ but given the small dietary requirement for molybdenum,^47^ and the limited extent to which dietary sulfur intake varies in young children, the relevance of diet to clinical presentation or diagnosis is questionable and likely only applies to mild cases of MoCD.^48^ Though some patients were enrolled who had a mild phenotype, the study was intended to evaluate patients with severe disease, and therefore data on diet were not collected.

## 5 CONCLUSION

Without disease‐modifying therapy, sulfite intoxication disorders are severe, rapidly progressive neurodegenerative disorders. Patients often present in the first neonatal days with seizures and feeding difficulties, rapidly develop significant neurologic disabilities, and have high mortality risk within the first 2 years after birth. Data from this natural history study, along with interventional data from the cPMP development program, will be important to further evaluate safety and efficacy for this therapy, which was recently approved in the United States.^41^

## CONFLICT OF INTERESTS

Ronen Spiegel was principal investigator for two clinical trials sponsored by Origin Biosciences, Inc. Bernd Schwahn has received travel reimbursement for attendance at a meeting sponsored by Origin Biosciences, Inc and was principal investigator for two clinical trials sponsored by Origin Biosciences, Inc. Liza Squires and Nils Confer are employees of, and shareholders in, Origin Biosciences, Inc.

## ETHICS STATEMENT

The study protocol and informed consent form were approved by the institutional review boards at each study site, and the study was conducted according to World Medical Association Declaration of Helsinki and International Council for Harmonization (ICH) guidelines. ClinicalTrials.gov Identifier: NCT01735188.

## Supporting information


**Appendix** S1: Supporting Information

## Data Availability

The methodology for this study is available at ClinicalTrials.gov (https://clinicaltrials.gov/ct2/show/NCT01735188). Further support for the data reported herein is available at http://origintx.com/posters.
