# Supplementary material for: Molybdenum cofactor deficiency: A natural history
Source: J Inherit Metab Dis. 2022 Mar 3;45(3):456–69. doi: 10.1002/jimd.12488 (PMC9313850; doi:10.1002/jimd.12488)
Supplement: Supplementary file 1 — Appendix S1: Supporting Information [file JIMD-45-456-s001.docx]

# SUPPLEMENTARY MATERIAL

## Supplementary Methods

### Patient identification

Because the disease is ultrarare and often misdiagnosed as hypoxic ischemic encephalopathy, the study used atypical strategies to recruit participants. Centers or physicians were approached if they were known to have a patient protocol for MoCD or ISOD, if a potential case was verbally communicated to a study investigator, or if they had previously published case reports of sulfite intoxication associated with MoCD/ISOD mutations (ie, *MOCS1, MOCS2, MOCS3, GPHN*, *SUOX*) or biochemical elevation in sulfite, xanthine, or S-sulfocysteine (SSC) and decreased urate. Letters were also sent to state newborn screening centers in the United States (US) to identify eligible patients.

### Genetic analysis

Genotype of MoCD was collected, if available. Genetic analysis was performed at each local site according to their own methodology and was considered retrospective. If a patient with a compound heterozygous mutation had post-neonatal onset and one of the mutations was also identified in a patient with neonatal onset, that mutation was considered to be associated with neonatal onset, while the other was considered associated with post-neonatal onset.

### Biomarker analysis

Prospective biomarker data were analyzed by a single central laboratory (Pharmaceutical Product Development, LLC; Wilmington, NC) using a validated method, and are presented within this manuscript. Previous reports have described methodology for these biomarker assessments.^25,S1,S2^ Briefly, dipotassium EDTA was added to plasma samples. Separate dilutions were made for SSC, xanthine, and urate, then acidified with 25 µL of 4:96 formic acid/water vol/vol. Protein was precipitated by adding 100 µL methanol followed by 500 µL acetonitrile. To measure urate, 50 µL supernatant was transferred to a 96-well plate and diluted with 1.00 mL of 75:25 acetonitrile/water vol/vol. For SSC and xanthine, 50 µL of supernatant was transferred to a 96-well plate and diluted with 200 µL acetonitrile. For urine SSC and xanthine samples, a 100-µL matrix aliquot was fortified with 100 µL of internal standard working solution 1, then a 25.0-µL aliquot was further diluted with acetonitrile. To analyze urine urate and creatinine samples, a 50.0-µL aliquot was added to water with internal standard working solution 1, vortexed and mixed, diluted serially with water and 75:25 acetonitrile/water vol/vol, and plated. Finally, all plasma samples and urine SSC and xanthine samples were injected into the HPLC column (Betasil Silica-100, 3 mm x 150 mm, 5 µm; Thermo Scientific, Waltham, MA) with MS/MS detection using negative ion electrospray (API 4000 Triple quadrupole LC-MS/MS; Sciex, Framingham, MA). Urine biomarker data were normalized to creatinine and reported relative to published reference ranges.^22^

References:

S1. Belaidi AA, Arjune S, Santamaria-Araujo JA, Sass JO, Schwarz G. Molybdenum cofactor deficiency: a new HPLC method for fast quantification of s-sulfocysteine in urine and serum. *JIMD Rep.* 2012;5:35-43.

S2. Pitt JJ, Eggington, M., Kahler, S. G. Comprehensive screening of urine samples for inborn errors of metabolism by electrospray tandem mass spectrometry. *Clin Chem.* 2002;48:1970-1980.

**Supplementary Figure 1**. Patients enrolled by country


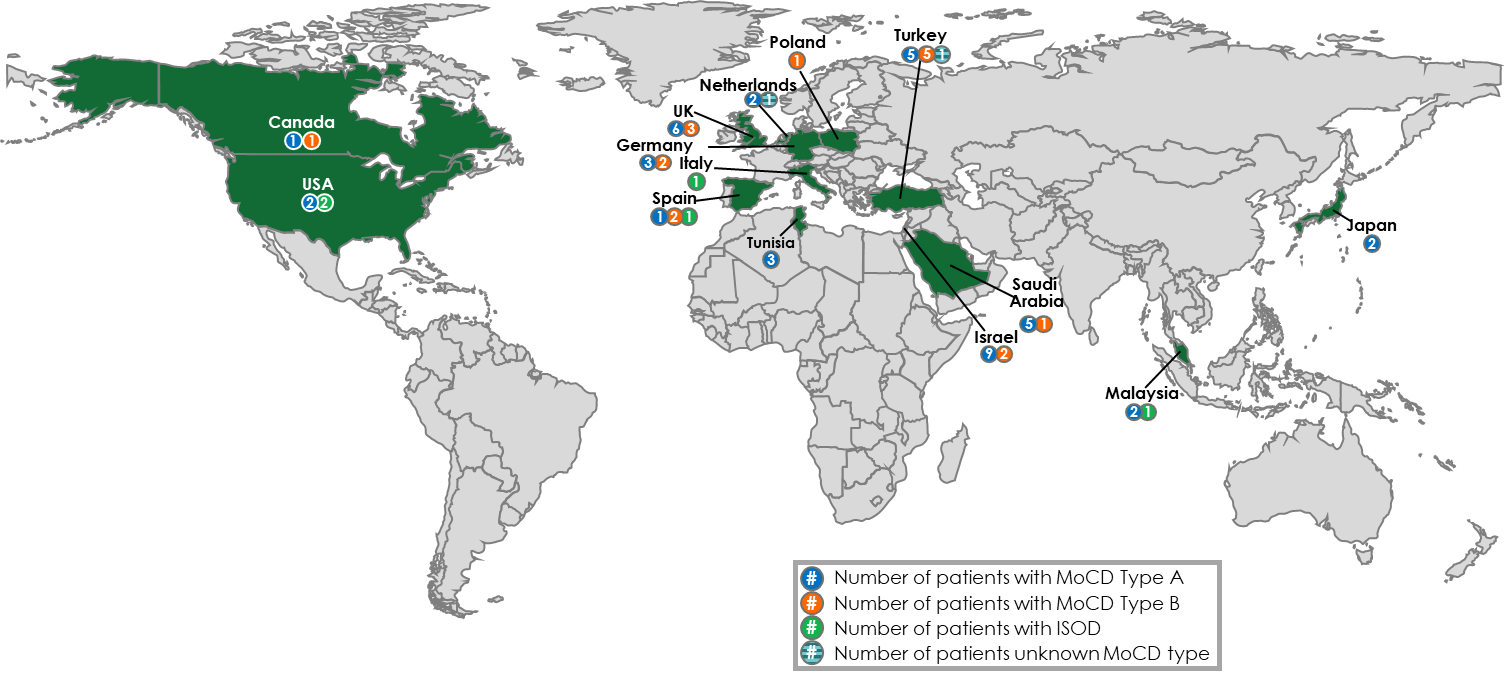


**Supplementary Figure 2.** Patient disposition
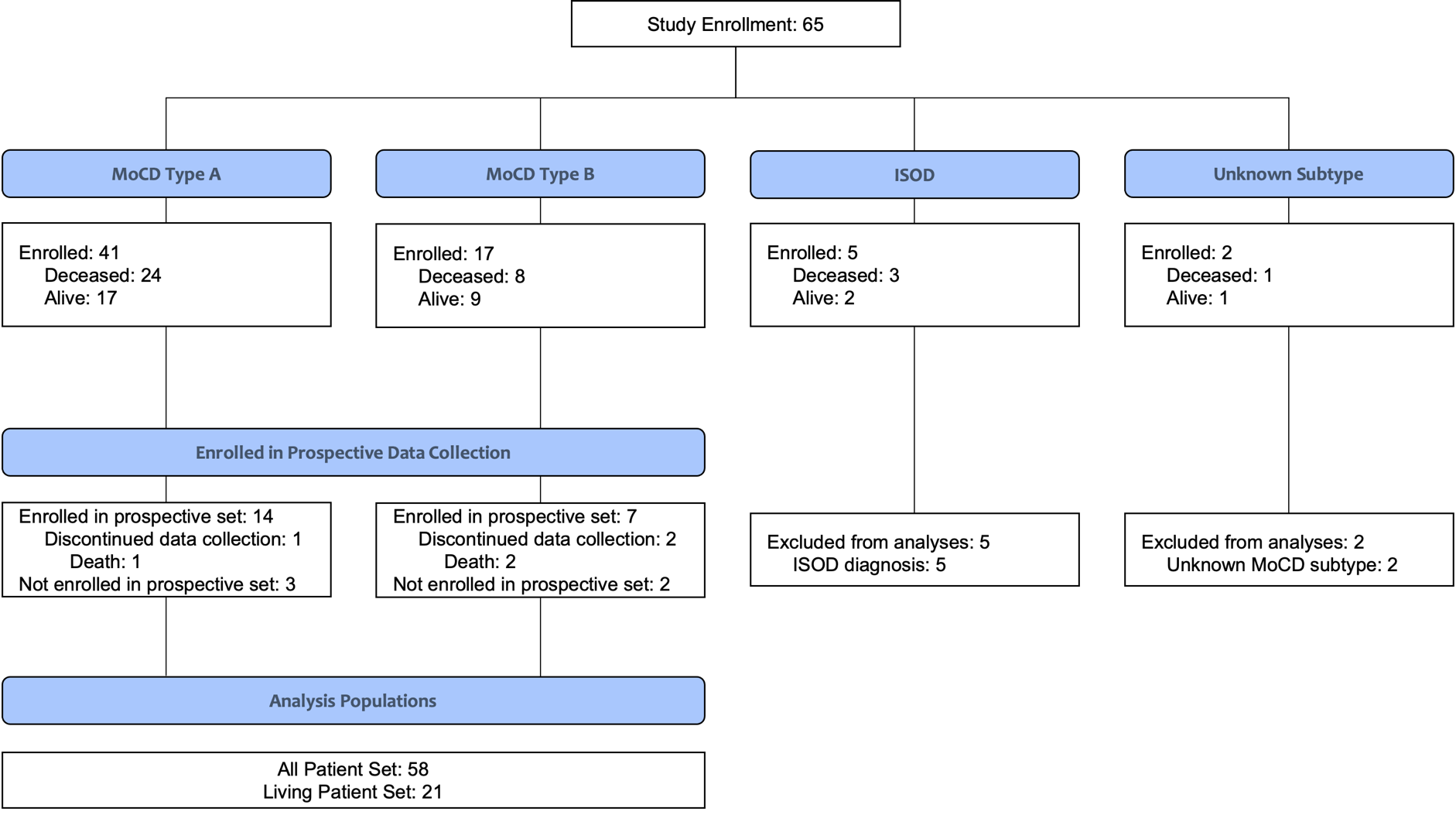


MoCD-A, molybdenum cofactor deficiency type A; MoCD-B, molybdenum cofactor deficiency type B.

“Living” means alive at last observation with current status unknown.

**Supplementary Figure 3**. Map of *MOCS1* and *MOCS2* genes and mutations identified in this study


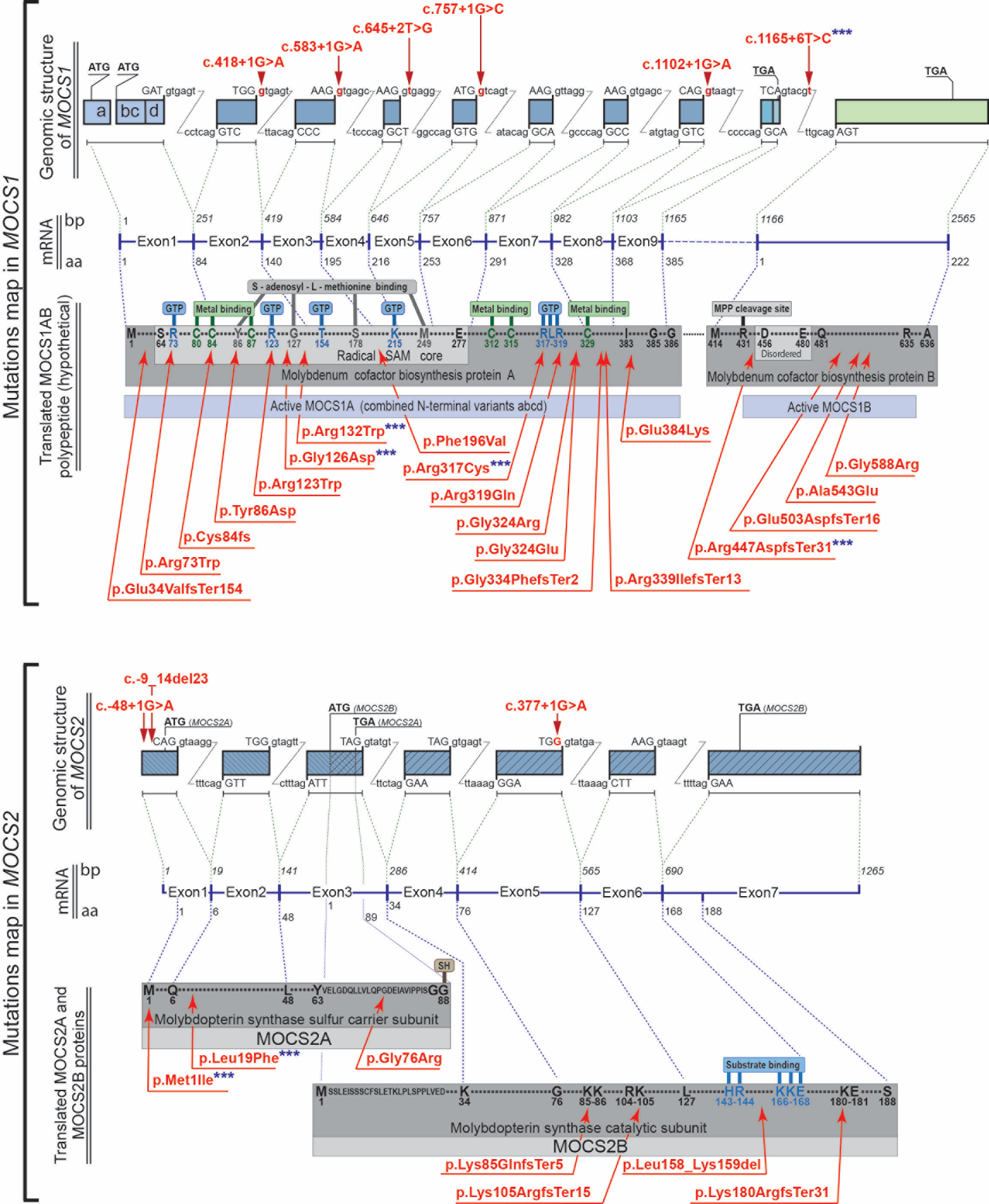


Note: For the *MOCS2* c.-9_14del23 mutation, there is no protein product (p.(0)) and therefore it is not presented at the protein level in this figure.

***Pathogenic variants associated with post-neonatal onset and/or attenuated progression.

**Supplementary Table 1**. MoCD sequelae

| **Characteristic, n (%)** | **Type A  n = 41** | **Type B  n = 17** | **Total^a^ N = 58** |
| --- | --- | --- | --- |
| Patients with any MoCD sequelae | 39 (95.1) | 15 (88.2) | 54 (93.1) |
| Limb hypertonia | 36 (87.8) | 13 (76.5) | 49 (84.5) |
| Developmental delay | 35 (85.4) | 12 (70.6) | 47 (81.0) |
| Truncal hypotonia | 29 (70.7) | 14 (82.4) | 43 (74.1) |
| Microcephaly | 26 (63.4) | 11 (64.7) | 37 (63.8) |
| Dysmorphic features | 26 (63.4) | 13 (76.5) | 39 (67.2) |
| Spastic quadriplegia | 23 (56.1) | 10 (58.8) | 33 (56.9) |
| Myoclonus | 22 (53.7) | 4 (23.5) | 26 (44.8) |
| Opisthotonos | 16 (39.0) | 8 (47.1) | 24 (41.4) |
| Cortical blindness | 17 (41.5) | 4 (23.5) | 21 (36.2) |
| Spastic diplegia | 11 (26.8) | 3 (17.6) | 14 (24.1) |
| Nystagmus | 10 (24.4) | 3 (17.6) | 13 (22.4) |
| Ectopic lenses | 9 (22.0) | 4 (23.5) | 13 (22.4) |
| Enophthalmos | 9 (22.0) | 2 (11.8) | 11 (19.0) |
| Stroke-like episodes | 4 (9.8) | 0 (0) | 4 (6.9) |

MoCD-A, molybdenum cofactor deficiency type A; MoCD-B, molybdenum cofactor deficiency type B.

Percentage calculated based on total population.

^a^MoCD sequelae were not reported for 2 patients with MoCD-A and 2 with MoCD-B.

**Supplementary Table 2**. Additional patient characteristics

| **MoCD Type** | **Genotype** | **Age at Disease Onset, days** | **MoCD Symptoms Reported** | **Provided Prospective Biomarker Data?** |
| --- | --- | --- | --- | --- |
| A | Unknown | 7 | Feeding difficulties  Seizures  Exaggerated startle response  Other | No |
| A | Unknown | 2 | Feeding difficulties  Seizures  Exaggerated startle response | No |
| A | Unknown | 1 | Feeding difficulties  Seizures  Other | No |
| A | Unknown | 1 | Feeding difficulties  Seizures  Intracranial hemorrhage | No |
| A | c.583+1G>A/c.583+1G>A | 6 | Feeding difficulties  Seizures  High-pitched cry | No |
| A | c.1338delG/c.1338delG | 1 | Other | No |
| A | c.1508_1509delAG/c.1508_1509delAG | 2 | Feeding difficulties  Seizures  Exaggerated startle response  High-pitched cry | No |
| A | c.256T>G/c.1150G>A | 1 | Feeding difficulties  Seizures  Exaggerated startle response  High-pitched cry  Other | No |
| A | c.970G>A/c.1150G>A | 2 | Feeding difficulties  Seizures  High-pitched cry  Metabolic acidosis | No |
| A | c.956G>A/c.956G>A | 17 | Feeding difficulties  Seizures  Exaggerated startle response  High-pitched cry | No |
| A | c.99_100delGG/c.99_100delGG | 6 | Feeding difficulties  Seizures  Exaggerated startle response | No |
| A | c.1165+6T>C/c.1165+6T>C | 927 | Other | Yes |
| A | c.1762G>A/c.1762G>A | 1 | Feeding difficulties  Other | No |
| A | c.1015_1018delCGGG/c.1150G>A | 1 | Feeding difficulties  Seizures  Other | No |
| A | c.251_418del/c.251_418del | 2 | Seizures  Exaggerated startle response  High-pitched cry | No |
| A | c.971G>A/c.971G>A | 1 | Feeding difficulties  Seizures  Exaggerated startle response  Metabolic acidosis | No |
| A | c.971G>A/c.971G>A | 4 | Feeding difficulties  Seizures  Exaggerated startle response  High-pitched cry  Metabolic acidosis | No |
| A | c.971G>A/c.971G>A | 1 | Feeding difficulties  Seizures  Exaggerated startle response  High-pitched cry | No |
| A | c.367C>T/c.367C>T | 1 | Feeding difficulties  Seizures  Exaggerated startle response | No |
| A | c.367C>T/c.367C>T | 1 | Feeding difficulties  Seizures  High-pitched cry | No |
| A | c.586A>G/c.586A>G | 1 | Feeding difficulties  Seizures  High-pitched cry | Yes |
| A | c.1643C>A/c.1643C>A | 1 | Feeding difficulties  Seizures  Intracranial hemorrhage | Yes |
| A | c.1643C>A/c.1643C>A | 2 | Seizures | Yes |
| A | c.394C>T/c.1000dupT | 46 | Feeding difficulties  Seizures  Exaggerated startle response  Other | No |
| A | c.394C>T/c.1000dupT | 3 | Feeding difficulties  Seizures | Yes |
| A | c.418+1G>A/c.418+1G>A | 3 | Feeding difficulties  Seizures | No |
| A | c.949C>T/c.949C>T | 222 | Feeding difficulties  Seizures | Yes |
| A | c.217C>T/c.217C>T | 2 | Feeding difficulties  Seizures  High-pitched cry | Yes |
| A | c.217C>T/c.217C>T | 1 | Feeding difficulties  Seizures  High-pitched cry  Other | Yes |
| A | c.757+1G>C/c.757+1G>C | 6 | Feeding difficulties  Seizures  Other | No |
| A | c.757+1G>C/c.757+1G>C | 16 | Feeding difficulties  Seizures | No |
| A | c.645+2T>G/c.645+2T>G | 2 | Feeding difficulties  Seizures | No |
| A | c.645+2T>G/c.645+2T>G | 2 | Feeding difficulties  Seizures  Exaggerated startle response  High-pitched cry  Metabolic acidosis  Other | Yes |
| A | c.1102+1G>A/c.1102+1G>A | 4 | Feeding difficulties  Seizures  Exaggerated startle response  High-pitched cry  Metabolic acidosis  Other | Yes |
| A | c.367C>T/c.367C>T | 3 | Feeding difficulties  Seizures  Metabolic acidosis | Yes |
| A | ^a^ | 1 | Feeding difficulties  Seizures  Intracranial hemorrhage | No |
| A | c.217C>T/c.217C>T | 1 | Seizures  High-pitched cry | No |
| A | c.217C>T/c.217C>T | 2 | Feeding difficulties  Seizures | Yes |
| A | c.1508_1509delAG/c.1508_1509delAG | 1 | Feeding difficulties  Seizures  Metabolic acidosis | No |
| A | c.217C>T/c.217C>T | 11 | Feeding difficulties  Seizures  High-pitched cry  Other | Yes |
| A | c.377G>A/c.1102+1G>A | 733 | Seizures | Yes |
| B | ^a^ | 1 | Feeding difficulties  Seizures  Exaggerated startle response  Other | Yes |
| B | c.539_540delAA/c.377+1G>A | 14 | Feeding difficulties  Seizures | No |
| B | Unknown | 22 | Feeding difficulties  Seizures  Exaggerated startle response  Other | No |
| B | c.226G>A/c.226G>A | 1 | Feeding difficulties  Seizures  Exaggerated startle response  High-pitched cry  Metabolic acidosis  Intracranial hemorrhage  Other | Yes |
| B | c.226G>A/c.226G>A | 6 | Feeding difficulties  Seizures  Exaggerated startle response  Other | No |
| B | c.57A>T/c.57A>T | Unknown | No symptoms recorded | Yes |
| B | c.-9_14del23/c.-9_14del23 | 5 | Feeding difficulties  Seizures  Metabolic acidosis  Other | No |
| B | c.226G>A/c.226G>A | 1 | Feeding difficulties  Seizures  Exaggerated startle response  High-pitched cry  Other | Yes |
| B | c.226G>A/c.226G>A | 2 | Feeding difficulties  Seizures  Metabolic acidosis  Other | No |
| B | c.3G>A/c.3G>A | 218 | Feeding difficulties  Seizures | No |
| B | c.-48+1G>A/c.-48+1G>A | 27 | Seizures  High-pitched cry | No |
| B | c.539_540delAA/c.539_540delAA | 2 | Feeding difficulties  Seizures  Intracranial hemorrhage  Other | No |
| B | c.252_253InsC/c.252_253InsC | 4 | Feeding difficulties  Seizures | Yes |
| B | c.3G>A/c.3G>A | 144 | Seizures | Yes |
| B | c.314delA/c.314delA | 27 | Feeding difficulties  Seizures  High-pitched cry | Yes |
| B | c.471_477delTTTAAAAinsG/  c.471_477delTTTAAAAinsG | 1 | Feeding difficulties  Seizures  High-pitched cry | No |
| B | c.3G>A/c.3G>A | 153 | Seizures  High-pitched cry | No |

MoCD-A, molybdenum cofactor deficiency type A; MoCD-B, molybdenum cofactor deficiency type B.

Percentage calculated based on total population.

Unknown: Genotype was unknown for 5 patients who died before study start without genetic testing. Four of these patients were considered to have MoCD-A because younger siblings were enrolled who had genetic confirmation of MoCD-A. The fifth patient was considered to have MoCD-B because both parents were carriers of the familial disease-causing variant in *MOCS2*.

^a^Two genetic reports communicated by local investigators were inconsistent with the reported disease subtype. We were not able to verify the genetic data and classified the patients according to the reported disease type.
